# Multimedia Input and Bilingual Children’s Language Learning

**DOI:** 10.3389/fpsyg.2020.02023

**Published:** 2020-08-12

**Authors:** He Sun, Bin Yin

**Affiliations:** ^1^National Institute of Education, Nanyang Technological University, Singapore, Singapore; ^2^School of Audiology and Speech Sciences, University of British Columbia, Vancouver, BC, Canada

**Keywords:** multimedia input, bilingual children, heritage language maintenance, input quantity, input quality

## Abstract

The current study seeks to explore the impact of multimedia input at home on bilingual children’s language outcomes. Two hundred and two Singaporean English-Mandarin kindergarteners’ multimedia experience (i.e., the resources and the amount of multimedia input) and conventional language exposure (e.g., language use with family members) were investigated with a parental questionnaire. A series of English and Mandarin tests were conducted to assess children’s proficiency (i.e., in receptive vocabulary, receptive grammar, verbal fluency) by standardized measures. Results demonstrated that the diversity of multimedia input is more important than the amount of multimedia input in promoting children’s Mandarin language maintenance, while controlling for children’s conventional language exposure, SES, and language aptitude. The number of multimedia sources is significantly and positively related to children’s general Mandarin proficiency. In contrast, English multimedia exposure at home exerts little impact on children’s general English proficiency. The findings indicate the unique contribution of multimedia diversity to children’s early heritage language maintenance. The strong social relevance of the study is discussed at the end of the paper.

## Introduction

### Multimedia Input and Child Bilingual Language Development

Input is considered crucial in bilingual children’s language development (Grüter and Paradis, 2014). The quantity and quality of input have been found to influence child bilingual’s route and rate of vocabulary and grammar acquisition^1^ (e.g., Paradis, 2011; Sun et al., 2018b). Researchers in child bilingualism tend to operationalize input quantity as the length of language exposure and the amount of daily communication in the given language with families, friends or in community settings (Unsworth, 2013). In terms of input quality, the emphasis has been on the richness, diversity and authenticity of input resources in children’s early literacy environment (Sun, 2019). Previous studies tend to focus on the impact of active communications (e.g., with family members) on bilingual children’s language development, and very few studies explore the influence of input from the multimedia perspective. We focus on the role of multimedia in children’s bilingual development in the current study.

The rapid emergence of multimedia such as content delivered through computers, tablets, and electronic books has substantially reshaped bilingual children’s input environment (Sun et al., 2019). Thus, the conventional input assessment that addresses children’s active communication with other interlocutors might not be able to holistically capture children’s daily input patterns. Multimedia could be generally defined as digital technologies combining various media such as video, audio, and text options (Chandler and Munday, 2011). It has been incorporated into almost every aspect of our lives, turning children into multimedia savvy users at a young age. For instance, a US study (Rideout, 2014) showed that around 80% of the participating children use educational media that are offered on TV, computer, and mobile devices at least once a week, among whom one-third use the service every day. A similar trend could be observed in Singapore, where children below 7 years old were found to frequently use smartphones, touchscreen tablets, and laptop computers (Ebbeck et al., 2016). By investigating 1058 parents’ and caregivers’ views of their children’s access to and time spent on multimedia devices, Ebbeck et al. (2016) demonstrated that children between 1 and 7 years of age used multimedia every day. In particular, 3-years-old spent the longest time on smartphones (*M* = 0.6 h per day), while 5-years-old spent the longest time using touchscreen tablets (*M* = 0.6 h per day).

Given such prevalent use of multimedia among young children, it would be pertinent to examine whether such media consumption might impact their language development. Indeed, multimedia has been found to positively influence early language learning, especially among monolingual kindergarteners (Rice et al., 1990; Singer and Singer, 1998), as well as for teenagers or adults in foreign language/bilingual settings (e.g., Cho and Krashen, 2000; Kuppens, 2010). The reason for this advantage may have to do with the dual coding theory of learning. It has been postulated that deeper learning occurs when information is presented in both the verbal (e.g., oral narration) and non-verbal modalities (e.g., dynamic visualization). The closer temporal alignment between the presentation of verbal and non-verbal information, the better the learning outcome is (e.g., Mayer, 2005). In this respect, multimedia materials provide non-verbal information that facilitates language comprehension by visualizing narrations, stories, or events in a congruent way (Jared et al., 2013; Wong and Samudra, 2019). In addition, multimedia content tend to be more engaging for children (Takacs et al., 2015), which contributes to their language learning. The bulk of research on the effect of multimedia in language learning comes from the monolingual literature, or research on older bilinguals. It remains to be seen the extent to which bilingual kindergarteners might benefit from multimedia input in their language learning.

Limited existing research suggests that in comparison to societal dominant languages, multimedia might have a more visible impact on bilingual children’s heritage language learning, because such children might have limited resources to receive heritage language input from the conventional channels. Take Singapore as an example where the overall sociolinguistic environment leads to an imbalance of input for English vs. non-English languages. Since 1965, English has been taken as the societal dominant language to facilitate inter-ethnic communication and business with the world. Official heritage languages (i.e., Mandarin, Malay, and Tamil), on the other hand, have been used to transmit heritage values and maintain cultural and racial identity. In schools, heritage languages are taught only as a subject, whereas English is used as the medium of instruction. This language policy has led to a substantial change in the home language environment due to parents’ utilitarian focus on English. According to Singapore Ministry of Education, 61% of children from the primary school cohort in 2011 were from English dominant families (Goh and Ng, 2015), and the percentage is probably higher based on recent large-scale investigations (e.g., Sun et al., 2018b). While children can access abundant and high-quality content in English, input for heritage languages tends to be more impoverished in quantity and quality (e.g., Sun et al., 2018b). Thus, maintenance of heritage languages in such a context poses a challenge. Given the relative lack of access to conventional, interactive forms of input in heritage languages, one might wonder whether multimedia input may play an outsized role in heritage language maintenance. Indeed, it was found that media input quantity contributed to vocabulary outcomes in heritage languages, but not in English in Singapore (Sun et al., 2018b). We continue to investigate this question in the current study where we look at both the quantity and quality dimensions (i.e., diversity of the resources) of multimedia input, for the acquisition of English and a heritage language (Mandarin Chinese) in Singapore.

### The Amount of Multimedia Input and Children’s Early Language Development

Existing research focusing on multimedia as augmentations to story narration has generally shown that the amount of multimedia input is positively related to vocabulary acquisition. A meta-analysis study (Takacs et al., 2015) drawing on data from 2147 children in 43 studies found that technology-enhanced storybooks conferred a small, but significant additional benefit on expressive vocabulary and story comprehension. Importantly, the analysis conducted in that study revealed that multimedia was particularly beneficial for “disadvantaged” children including those from bilingual, immigrant backgrounds, low socioeconomic status (SES) families, and those with other at-risk characteristics. However, the focus of the meta-analysis in Takacs et al. (2015) was deliberately narrow – only studies that included (oral) story narrations were selected for analyses. While this was necessary to enable a valid comparison with the traditional book reading experience, it also meant that studies that looked at the general, incidental effects of multimedia on language and literacy were not included. We look at some of those studies below.

Studies on the incidental effects of multimedia on language and literacy among monolingual children appear to show that results depend on the age. For example, Singer and Singer (1998) found that preschoolers who had viewed ten preselected episodes of Barney and Friends showed significant vocabulary gains. Another study (Rice et al., 1990) found that children who had been frequent viewers of *Sesame Street* performed significantly better on vocabulary tests at age 5 than children in a comparison group. On the other hand, studies focusing on younger monolinguals (around 3 years of age and below) did not yield positive results for the effects of multimedia exposure (e.g., Alloway et al., 2014; Taylor et al., 2018). For example, in a study on 131 British children aged 6–36 months, Taylor et al. (2018) found no effect of screen time (TV or mobile devices) on the vocabulary knowledge of the children as measured by parental reports. Alloway et al. (2014) likewise relied on parental reports as a measure of 30 British toddlers’ vocabulary and failed to detect any effects of TV watching on children’s language development.

In bilingual and foreign language settings, the effects of multimedia exposure on incidental vocabulary learning are likewise mixed. Kuppens (2010) looked at incidental foreign language learning (English) among Flemish Dutch-speaking pupils (around 11 years old). These pupils had never been formally taught English in their school curriculum, but had access to a wide range of English-language media in their daily life. It was found that subtitled TV/movies, and computer games had significant effects on translation scores between Dutch and English for these students. In addition, the more time spent on those multimedia resources, the better their translation scores were. In heritage maintenance, Cho and Krashen (2000) reported that for ethnic-Korean adults who had arrived in the United States at an early age, watching television in Korean (regardless of the program) was a significant predictor of self-reported Korean language proficiency. Sun et al. (2020) likewise found that the amount of heritage language media input at home was a significant predictor of Singaporean heritage learners’ receptive vocabulary. On the other hand, Scheele et al. (2010) did not find any significant correlation between the frequency of watching educational TV program in the heritage language and vocabulary development in Moroccan-Dutch and Turkish-Dutch 3-years old. Another study showing null results was Patterson (2002) who found that television viewing did not predict vocabulary size for either Spanish or English in the bilingual toddlers (around 2 years of age) they studied. The results of these studies seem to mirror those from the monolingual literature in showing that older children were more likely to benefit from multimedia exposure than younger children.

### The Resources of Multimedia Input and Children’s Language Development

In addition to the role of input amount, it is also relevant to look at the issue of the quality of multimedia in predicting language outcome. Broadly, this question relates to the issue of input quality in language learning which can be defined as “variation in experience with native-speaker input, rich and complex input gained through activities like reading” (Paradis, 2011, p. 217). Researchers have found that input quality is important for bilinguals’ language and literacy acquisition. For example, Scheele et al. (2010) looked at enriching home language activities such as reading, story-telling, and educational TV among Moroccan-Dutch and Turkish-Dutch families in the Netherlands. Their study found a significant correlation between these quality-oriented activities in the L2 (Dutch) and L2 vocabulary outcomes. Paradis (2011) operationalized the notion of input quality in terms of mothers’ self-assessed proficiency (in L2 English), mothers’ education, and richness of the English environment outside school. Her research revealed that richness of the English environment was a significant predictor for vocabulary scores of her 169 child bilingual participants. Sun et al. (2020)’s research on Singaporean children’s receptive vocabulary included measures on input quality such as book reading, and their analyses showed that those were significant predictors of these 457 children’s receptive vocabulary growth.

We also take up the issue of the quality of input in children’s multimedia exposure. Prior research has touched on the issue of different sources of enriching language activities (e.g., reading, story-telling, TV/movies). In addition, there is recent though limited evidence suggesting that the notion of variety in a given literacy activity contributes to learning outcomes. In a study on book-sharing interactions, Luo et al. (2020) examined the role of book variety in literacy outcomes of children from low-income, ethnic-minority homes in the United States. In the study, mothers of the children were asked to report whether they read each of 10 pre-specified types of books to their children, in addition to indicating the total number of books available at home. Those 10 types of books were divided into two categories for further analyses: concept books (number, colors, letters, shapes, and opposite concepts) and narrative books (daily activities, family relationships or friendships, religious or cultural beliefs, folk tales, and humor). The study found significant effects of book variety for both categories. Namely, the variety of narrative books explained children’s narrative contributions during book-sharing interactions whereas the variety of concept books predicted children’s referential contribution. The authors concluded by recommending a “varied diet of literacy resources” for literacy development (p. 229). Following this finding, we zoom in on the topic of multimedia exposure and explore the question whether the diversity of different types of multimedia alone contributes to early bilingual language acquisition, over and above the overall quantity of multimedia exposure. Specifically, parents were asked to indicate whether children were exposed to six sources of multimedia input: TV programs, Videos, Audio, Materials demonstrated via electronic devices, e-books, and computer games. We would like to find out whether being exposed to different varieties of these multimedia alone accounts for their language outcome. The rationale behind our thinking is that different types of multimedia may typically use different kinds of lexicon and grammar structures. For example, TV programs might expose children more to colloquial language and informal lexicon, whereas e-books are likely to contain more sophisticated vocabulary and grammar. The more variety of multimedia exposure therefore leads to a wider range of language input for the child. To the best of our knowledge, this question has not been directly addressed in the literature on multimedia exposure, and we would like to pursue it in the current study.

### Other Influential Factors in Bilingual Language Acquisition

A set of factors reflecting learners’ specific capability for language learning have also been found to influence bilingual children’s language learning (Carroll and Sapon, 2002). These factors are collectively known as language aptitude, and include components such as phonological short-term memory and non-verbal intelligence. Each of these contributes to bilingual children’s language learning in different ways (e.g., Knell et al., 2007; Alexiou, 2009; Paradis, 2011). Short-term memory facilitates word articulation and semantic memory by helping children retain the novel sequence of the phonological properties of a language (Gathercole and Baddeley, 1989; Paradis, 2011). Non-verbal intelligence enables a bottom-up approach to linguistic tasks whereby children are able to infer and reorganize structures and patterns (Hakuta and Diaz, 1985; Daller and Ongun, 2018).

Social-economic (SES) has also been found to affect bilingual children’s language. Usually measured through maternal education level and household income, SES has been shown to significantly predict bilingual children’s vocabulary and grammar acquisition (Blom et al., 2012; Sun et al., 2018b). This can be illustrated by the investment model, as the time and effort the parents spend on their children are equivalent to the number of potential avenues for promoting children’s language and cognitive development (Dickinson and Tabors, 2001; Hartas, 2011).

### The Current Study

The current study intends to explore the relationship between multimedia input and children’s bilingual language outcomes. In the current paper, multimedia exposure refers to TV programs, videos (e.g., movies via DVD player), audios (e.g., songs via CD player), ebooks, computer games, and other materials demonstrated via E-devices (e.g., apps on iPad). Before proceeding to the questions of the current study, it is important to provide a brief sketch of the overall linguistic environment for the child participants in the current study. As mentioned above, our children are heritage language learners in Singapore where English together with three ethnic languages (Mandarin Chinese, Malay and Tamil) function as official languages. Nevertheless, the predominance of English in various domains of life, including education, government, and inter-ethnic communication (e.g., Bokhorst-Heng, 1999) has resulted in a situation unfavorable to the acquisition and maintenance of heritage languages. Specifically, as described in Sun et al. (2018b), the linguistic environment in Singapore is considered input-poor for heritage languages as a result of the comparative lack of exposure to these languages in various spheres of life. In such a situation, the quantity and quality of input plays a differential role for the learning of heritage languages vs. the societal dominant language (English), as already demonstrated in prior research (e.g., Paradis, 2011; Sun et al., 2018b). With this in mind, there are two specific hypotheses we would make for our current study:

1 Multimedia input has a larger effect on children’s Mandarin learning than on English learning. It is because Mandarin has much less amount and resources of input from the conventional channels than that of English, and multimedia input could be an important supplement for children’s Mandarin exposure at home. Moreover, children’s heritage language may be weaker than their societal dominant language, and the features of multimedia input may scaffold children’s vocabulary and grammar learning in the weaker language development to a larger extent.
2 Both the number of resources and the amount of multimedia input are crucial in children’s language acquisition, as the former might provide the children with a higher quality of input (e.g., the diverse vocabulary) and the latter may offer children larger quantity of language exposure.

## Materials and Methods

### Participants

We recruited two hundred and two young English-Chinese bilingual preschoolers in Kindergarten 1 (4–5 years old; 89 boys and 112 girls) from 21 preschools in Singapore to participate in this study. The participants were recruited based on information provided by teachers and parents. The two selection criteria for participation were: firstly, children should be Mandarin-English bilingual language learners. Participants exposed to more than two languages at home or recent immigrant children from China were excluded^2^. Secondly, there should be no history of developmental delays or impairment. Children varied in social-economic status, but most of them were from middle-class families, with their family income well above the relative poverty line of the country (> S$2500, Donaldson et al., 2013). The average monthly household income was between S$7500 and S$7999. The parental questionnaire contained 20 income options, ranging from “Below 1,000” to “10,000 and over,” with S$500 increment for each higher level (*M* = 15.01, *SD* = 5.07, *range* = 0–19). On average, parents’ highest level of education was a polytechnic diploma or bachelor’s degree (e.g., mother’s education; *M* = 5.34, *SD* = 1.28, *range* = 2–8, ranging from “No qualification” to “Doctorate degree”).

### Data Collection and Measures

The first author of this paper obtained ethics clearance from the University’s institutional review board. Consent was obtained from parents through forms disseminated at kindergartens. Prior to test administration, children also provided their assent to complete the tasks. Children’s English and Mandarin competencies (i.e., vocabulary and grammar) and cognitive capacities were assessed with standardized measures, while a parental questionnaire was used to collect information related to their bilingual environment at home. The sections below provide further information about the measures and the questionnaire in the study.

#### English and Mandarin Vocabulary Breadth

To measure children’s English and Mandarin receptive vocabulary, we used the Bilingual Language Assessment Battery (BLAB) (Rickard-Liow et al., 2013), a standardized test modeled after the Peabody Picture Vocabulary Test II (Dunn and Dunn, 2007). The auditory-picture matching task was developed locally and has been reported to have good reliability in the context of Singapore within the original bilingual norming sample (Rickard-Liow et al., 2013). During the assessment, children were presented with four pictures while listening to recorded words in the program. They were then asked to select one picture out of the four that best conveyed the understanding of the word presented to them. The assessment consisted of 3 practice trials and 80 test trials in total.

#### English and Mandarin Vocabulary Depth

Children’s productive vocabulary depth was assessed via a verbal fluency task. Participants were asked to name as many English and Mandarin words as they could within a general theme in 1 min. The chosen topics included food and animals, as previous studies have shown their effectiveness in testing child bilinguals’ verbal fluency (e.g., Schwartz et al., 2012; Sun et al., 2018a). One point was awarded for an appropriate word. Higher scores indicate greater vocabulary depth in children.

#### English and Mandarin Grammar

The English Test for Reception of Grammar Version 2 (TROG; Bishop, 2003) and the Mandarin Grammar Receptive Test (MGRT; Sun, 2019) were used to assess children’s receptive grammar knowledge. Similar to the procedures in the BLAB, children were presented with four images and a spoken sentence at the same time. Their task was to select one image out of the four pictures based on their understanding of the sentence heard. There were 3 practice trials and 60 test trials in total. Both tests have demonstrated good external validity and internal reliability.

#### Non-verbal Intelligence

The Ravens Colored Progressive Matrices (CPM) test (Raven and Rust, 2004) was administered as a non-verbal measure of children’s general cognitive ability, and consists of three sections (A,AB,B) containing twelve items each. Children were provided with an incomplete puzzle and asked to choose one out of six pieces to complete the puzzle. The items are arranged to assess the consistency in the children’s reasoning using analogy and inference skills. The Ravens CPM test has been extensively used across a variety of settings worldwide as a culture-neutral instrument of non-verbal intelligence.

#### Phonological Working Memory

The digit span and non-word repetition sub-tests of the Comprehensive Test of Phonological Processing (CTOPP; Wagner et al., 1999) were administered to measure phonological short-term memory. The two tests comprise a list of digits or non-words in English, and participants were asked to repeat and pronounce what they heard on the computer. There were 21 and 18 trials respectively for each subtest, and each subsequent trial increased in difficulty as the length of the digits and non-words increased in length. The tests were terminated after five consecutive incorrect responses.

#### Parental Questionnaire

We administered a parental questionnaire that included items adapted from existing related studies to explore children’s conventional language exposure and literacy environment (Sun et al., 2016). Compared with the prior questionnaire, the current version was mainly concerned with children’s bilingual input environment at home. The questionnaire focused on children’s media usage (i.e., amount and diversity of multimedia input) and conventional language input at home. Children’s multimedia type and amount have been estimated by the total hours and numbers of sources that children were exposed in TV programs, videos, audios, eBooks, and computer games via digital devices per week. Home language input and output were measured by the amount of time family members and friends interacted with children in English and Mandarin. Children’s cumulative input has been estimated with their onset age to steadily receive English and Mandarin exposure. For home literacy environment, parents were asked about the number of English and Mandarin books at home using a scale ranging from 0 to 6 (0 = None, 1 = 1–10, 2 = 10–30, 3 = 30–60, 4 = 60–90, 5 = 90–120, 6 = More).

### Data Analysis

The authors used IBM SPSS AMOS 25 to build up structural equation modeling (SEM) for the postulated relationships in the two hypotheses. SEM refers to a modeling technique that allows the evaluation of multiple correlational and causal assumptions simultaneously. It has been widely applied in sociology, psychology, linguistics, and other social sciences to explore complex associations. According to the literature (Klem, 2000), four indexes are crucial to the evaluation of the model fit, including Chi-square, comparative fit index (CFI), Tucker and Lewis’s fit index (TLI), and the root mean square error of approximation (RMSEA). A non-significant Chi-square indicates a good model fit as it implies that the theoretical model and the data-driven model are not significantly different. Nevertheless, as Chi-square is sensitive to sample size, researchers may end up with a significant *p*-value for Chi-square easily. In contrast, TLI and CFI values are less affected by sample size. Higher TLI and CFI values (= 0.9) and lower RMSEA values (=0.06) indicate a good model fit (Kenny and McCoach, 2003). Approximately 3.76% of data were missing mainly due to parent’s overlooking of a survey item or children’s absence on the testing day due to illness. The authors used the Full-Information Maximum Likelihood method in AMOS to estimate the missing values.

## Results

### Descriptive Statistics and Bivariate Correlations

Table 1 presents the descriptive statistics of 202 children’s home language environment (i.e., multimedia input and conventional language input), social-economic status (i.e., mother’s education level, and household income), language aptitude (i.e., phonological short-term memory, and non-verbal intelligence), and bilingual language skills (i.e., vocabulary breadth, vocabulary depth, and grammar). The results [i.e., the standard deviation (SD) and range figures in Table 1] indicate that group-wise, children’s bilingual home language environment and learning outcomes varied substantially. Take children’s multimedia time as an example, some children could receive as much as 92 h of English input per week, while some children have no English multimedia input at all. The substantial variation among children’s learning environment and language proficiency yielded high SD figures (e.g., the SD of children’s multimedia time is 17.22). Children’s bilingual language environment and outcomes are not only different from each other, but also within each child’s dual languages. Paired sample *t*-tests demonstrated that Singaporean children’s English environment is significantly and systematically better than their Mandarin environment. Except for the onset age of having steady English and Mandarin input at home, children have significantly more English input than Mandarin input from multimedia and family members. They have a significantly larger number of multimedia resources and books in English than in Mandarin. They also use English significantly more often with their family members. Regarding children’s language outcomes, their English vocabulary breadth, vocabulary depth, and grammar were substantially better than their skills in Mandarin. The dominance of English in children’s language environment and outcomes keep in line with the previous findings (e.g., Sun et al., 2018b), confirming our assumption of the unbalanced situation of children’s bilingual language learning in Singapore. As home input and output were highly correlated in English and Mandarin languages respectively (i.e., *r* = 0.874 in English, *r* = 0.879 in Mandarin), we averaged home input and output in each language and used it to reflect the frequency of family members’ and children’s interaction in that language.

**TABLE 1:** Descriptive statistics and paired *t*-tests of the bilingual language environment and learning outcomes.

	English			Mandarin			Paired *t-*test	
			
	N	*M* (*SD*)	Range	*N*	*M* (*SD*)	Range	*t*	*p*
Multimedia type	201	2.27 (1.17)	0–6	201	1.26 (1.31)	0–6	10.49	0.00
Multimedia time	196	23.01 (17.22)	0–92	199	10.62 (13.75)	0–56	8.70	0.00
Home input	199	3.01 (2.30)	0–13.71	201	2.39 (2.17)	0–12	2.60	0.01
Home output	198	2.57 (2.02)	0–11.89	199	2.04 (2.09)	0–9.6	2.32	0.02
Onset age	199	16.43 (14.47)	0–61	202	16.89 (14.82)	0–61	−0.57	0.57
Book number	201	2.53 (1.34)	0–6	202	1.94 (1.33)	0–6	6.76	0.00
Vocabulary breadth	180	43.41 (8.05)	24–64	191	34.68 (9.99)	7–61	9.67	0.00
Vocabulary depth	182	14.83 (5.02)	0–31	189	7.43 (5.05)	0–25	15.01	0.00
Grammar	180	41.31 (15.99)	0–70	189	35.97 (11.07)	9–57	8.89	0.00
Mother education	200	5.18 (1.29)	2–8					
Household income	198	14.25 (5.57)	2–20					
Phonological memory	189	19.90 (4.51)	6–31					
Non-verbal intelligence	181	20.17 (4.88)	8–33					

*Multimedia Type, the numbers of multimedia resources that children obtain English and Mandarin input per week; Multimedia Time, the numbers of hours that children spend on multimedia in English and Mandarin per week; Home Input, the numbers of hours that family members on average speak to children in English and Mandarin per day; Home Output, the numbers of hours on average that children speak to family members in English and Mandarin per day; Onset Age, the ages that children start to receive consistent and significant exposure to English and Mandarin; Book Number, the numbers of English and Mandarin books at home on a 1–7 point scale; Vocabulary Breadth, English and Mandarin receptive vocabulary size measured by BLAB; Vocabulary Depth, English and Mandarin productive vocabulary fluency; Grammar, English and Mandarin receptive grammar measured by MGRT and TROG; Mother Education, mothers’ highest educational level; Household Income, monthly family income on a 1–20 increasing point scale; Phonological Memory, short-term phonological memory score based on digit span and non-word repetition; Non-verbal Intelligence, non-verbal IQ score as a measure of analytic reasoning using Raven’s (Sun et al., 2016).*

### Multimedia Input and Early Mandarin Language Skills

Mandarin vocabulary breadth, vocabulary depth, and receptive grammar were used to create the latent “Mandarin” factor, and CFA was performed to measure the fitness of the latent factor. The results of maximum likelihood estimation indicated that the assumption for the latent factor holds, X^2^(3) = 253.610, *p* < 0.001, CFI = 1, TLI = 1, RMSEA = 0.00, as CFI, TLI, and RMSEA were consistent with the cutoff model-fit criteria recommended by previous studies (e.g., Kenny and McCoach, 2003), indicating a reasonable factor structure of our model.

The association between children’s Mandarin language environment and their Mandarin outcomes (i.e., vocabulary and grammar) have been demonstrated in Table 2. Children’s general Mandarin proficiency was predicted by the multimedia exposure in Mandarin language at home (i.e., multimedia type, and multimedia time), conventional exposure in Mandarin language at home (i.e., the average hours of Mandarin use between family members and the child per day, children’s onset age of having steady Mandarin input, and the number of Mandarin books at home), and individual differences in familial social-economic status and language aptitude (i.e., mother’s educational level, household income, phonological memory, and non-erbal intelligence). The results indicated that it is the number of multimedia resources but not the amount of multimedia input in Mandarin that significantly predicted children’s better Mandarin outcomes. Diverse multimedia input was positively and significantly related to children’s general Mandarin skills. Conventional environmental factors, such as Mandarin input and output in the current family environment, the onset age of having steady Mandarin input, and the number of Mandarin books at home, were also positively and significantly associated with children’s general Mandarin proficiency. Besides the multimedia and conventional language input at home, children’s language aptitude mattered. Children with better short-term phonological memory and non-verbal intelligence tended to have better Mandarin proficiency. The SEM model explained 47% of the variance in children’s Mandarin proficiency, with good model-fit statistics [X^2^(18) = 29.738, *p* = 0.04, CFI = 0.98, TLI = 0.913, RMSEA = 0.057].

**TABLE 2:** Results of structural equation modeling on Mandarin language outcomes.

	**Path**	***B***	***SE***	**β**	**C.R.**	***P***
Multimedia language environment	Man. Multimedia Type – > Mandarin	1.24	0.56	0.18	2.20	0.03*
	Man. Multimedia time– > Mandarin	−0.06	0.05	−0.09	−1.09	0.28
Conventional language environment	Man. Home use – > Mandarin	1.14	0.30	0.26	3.83	***
	Man. Onset age – > Mandarin	−0.14	0.05	−0.19	−3.00	**
	Man. Book number – > Mandarin	1.52	0.45	0.23	3.40	***
Other control factors	Mother education – > Mandarin	0.33	0.51	0.05	0.64	0.53
	Household income– > Mandarin	0.04	0.12	0.02	0.30	0.76
	Phonological memory – > Mandarin	0.66	0.13	0.33	5.09	***
	Non–verbal intelligence – > Mandarin	0.41	0.12	0.23	3.44	***
Mandarin latent factor	Mandarin – > Man. Vocabulary breadth	1.00		0.89		
	Mandarin – > Man. Vocabulary depth	0.41	0.04	0.72	11.15	***
	Mandarin – > Man. Grammar	1.06	0.08	0.84	13.45	***

***p < 0.001, **p < 0.01, *p < 0.05. X^2^(18) = 29.738, p = 0.04, CFI = 0.98, TLI = 0.913, RMSEA = 0.057. B refers to estimate of unstandardized regression coefficients/weights, SE refers to approximate standard error, β refers to estimate of standardized regression coefficients/weights, and C.R. refers to critical ratio (t-value). Interested readers may refer to Finch et al. (2016) for details of the terminologies.

### Multimedia Input and Early English Language Skills

Similar to Mandarin language measures, English vocabulary breadth, vocabulary depth, and receptive grammar were used to create a latent “English” factor. The fitness of the latent factor has been examined with CFA, and the model fits are satisfying [X^2^(3) = 127.891, *p* < 0.001, CFI = 1, TLI = 1, RMSEA = 0.00].

The relationship between children’s English language input (via multimedia and conventional exposures) and their English skills has been summarized in Table 3. Children’s general English proficiency was predicted by their home English multimedia use (i.e., type and amount), conventional exposures related to English input at home (i.e., use with family members, age of onset, and the number of books in English), and other control variables (i.e., mother’s educational level, household income, phonological awareness, and non-verbal intelligence). Different from the Mandarin SEM results, neither the type nor the amount of English multimedia input significantly predicted children’s general English proficiency. Children’s English interaction with family members mattered. The more hours per day they used English, the better the children’s general English skills were. Besides, children’s language aptitude (i.e., phonological awareness and non-verbal intelligence) also significantly predicted children’s English language outcomes. In total, the model explained 51% of the variance in children’s general English proficiency. The model fits are consistent with the cutoff criteria recommended in the literature (i.e., CFI = 0.991, TLI = 0.962, RMSEA = 0.03).

**TABLE 3:** Results of structural equation modeling on English language outcomes.

	**Path**	**B**	**SE**	**β**	**C.R.**	**P**
Multimedia language environment	Eng. Media type – > English	−0.29	0.43	−0.05	−0.67	0.50
	Eng. Media time – > English	0.04	0.03	0.09	1.16	0.25
Conventional language environment	Eng. Home use – > English	0.52	0.23	0.16	2.22	0.03*
	Eng. Onset age – > English	−0.07	0.04	−0.13	−1.84	0.07
	Eng. Book number – > English	0.50	0.36	0.10	1.38	0.17
Other control factors	Mother education – > English	0.43	0.41	0.08	1.05	0.30
	Household income – > English	0.03	0.10	0.03	0.34	0.73
	Phonological memory – > English	0.56	0.11	0.38	5.33	***
	Non-verbal intelligence – > English	0.58	0.10	0.43	5.99	***
English latent factor	English – > Eng. vocabulary breadth	1.00		0.82		
	English – > Eng. vocabulary depth	0.34	0.06	0.45	5.63	***
	English – > Eng. grammar	1.87	0.20	0.78	9.35	***

***p < 0.001, *p < 0.05. X^2^(18) = 21.344, p = 0.262, CFI = 0.991, TLI = 0.962, RMSEA = 0.03. B refers to estimate of unstandardized regression coefficients/weights, SE refers to approximate standard error, β refers to estimate of standardized regression coefficients/weights, and C.R. refers to critical ratio (t−value). Interested readers may refer to Finch et al. (2016) for details of the terminologies.

## Discussion

The current study examined how multimedia language input might influence English-Mandarin bilingual children’s dual language skills, controlling for children’s conventional language input factors at home (i.e., language use, age of onset, and literacy environment), children’s family SES (i.e., mother’s educational level, and household income), and language aptitude (i.e., phonological short-term memory and non-verbal intelligence). Two specific hypotheses were raised based on the literature and the bilingual language environment in Singapore. We hypothesized that (1) children’s Mandarin learning (i.e., heritage language) might benefit more from multimedia exposure than their English learning (i.e., societal dominant language), and (2) both the number of multimedia resources and the amount of multimedia input would be significantly associated with children’s Mandarin learning. Our results confirmed the first hypothesis. It was children’s Mandarin performance but not English performance (i.e., language outcome factors based on children’s Mandarin/English vocabulary breadth, vocabulary depth, and grammar) that significantly related to children’s multimedia input. The contribution of multimedia input to children’s Mandarin language performance is unique, which is on top of the variance explained by conventional home language exposure and children’s language aptitude. In terms of our second hypothesis, we were able to confirm half of the assumption, as only the number of multimedia resources in Mandarin was significantly associated with children’s Mandarin performance. Our results contradicted some previous findings (e.g., Kuppens, 2010) that demonstrates the significance of multimedia quantity (operationalized as multimedia time in the current study) on children’s early language acquisition. In the following sections, we discuss our findings in relation to the two hypotheses respectively.

### The Differential Effects of Multimedia Input on Bilingual Children’s Language Learning

Previous studies found that Singapore children’s bilingual input environment is not balanced (Dixon et al., 2012; Sun et al., 2018b). They have an input-rich English environment while a relatively input-poor heritage language environment, at both the input quantity and input quality levels (Sun et al., 2018b). The results of the paired *t*-tests in our study were in line with the existing studies, and confirmed the advantage of children’s English environment in family-child interactions and literacy resources at home. In such a situation, multimedia offered children an important channel to receive additional language exposure, and this extra input might substantially promote children’s heritage language outcome (as in the current study). In contrast, children have ample English input from various interlocutors at home and in the community; therefore, the additional input from multimedia may exert much less influence on children’s English learning outcome, as the conventional environment has already provided children with the “critical mass” of input to develop their English language skills properly (Sun et al., 2018b).

Multimedia may not only increase children’s input quantity in heritage language, but also provide them with more comprehensive input thanks to the features of multimedia. Previous studies have shown that learners’ prior knowledge is crucial as it determines how learners would process the information by linking the unknown with their existing knowledge. Information that engages multiple channels might be beneficial for novice learners but redundant for expert learners according to The Expertise Reversal Principle (Kalyuga et al., 2012). Learners with higher language skills may more efficiently process the input, thus additional information presented in the multimedia material may be redundant and cause cognitive overload (Mayer, 2009). For learners with lower language skills, the animated and interactive language input might provide children with additional contextual cues to extract semantic and syntactic information from the input. Take storybook reading as an example. It is one of the most popular activities among children, and is assumed to provide a meaningful context for children to acquire unfamiliar words and grammar (Weizman and Snow, 2001). Nonetheless, children with limited language knowledge (e.g., Mandarin language learners in the current study) may benefit less from the reading activities, due to the gap between their language skills and those required for processing the narration. They may fail to derive the meaning of unknown words and grammar from the verbal context and consequently have difficulties in figuring out the story plots (Verhallen and Bus, 2010). Well-designed animated eBooks hold good promise for children’s emergent literacy in this case, as such books can stimulate readers’ visual, auditory and even kinesthetic senses to comprehend a story and unfamiliar language via the congruence between non-verbal sources (motion pictures, images, sound, and music) and the narration (Sun et al., 2019), as predicted by the dual coding theory of learning. The “enhanced” message could scaffold learners to pick up the target information more easily and establish a coherent mental representation. In the current study, children’s Mandarin skills are significantly lower than their English skills in all three aspects we have measured (vocabulary breadth, *t* = −9.67, *p* < 0.001; vocabulary breadth, *t* = −15.01, *p* < 0.001; grammar, *t* = 8.89, *p* < 0.001). Therefore, they might benefit from multimedia-powered Mandarin input to facilitate their language learning.

### The Number of Resources and Amount of Multimedia Input and Child Mandarin Learning

The exploration of the second hypothesis further narrows down the effective components of the multimedia input. Contrary to our prediction, only the number of resources but not the amount of multimedia input has been found to be significantly related to children’s general Mandarin competence. The non-significance of multimedia input *per se* might be due to the mismatch between children’s current language level and the complexity of some of the content presented via multimedia. Researchers have noted that certain conditions need to be met for multimedia resources (e.g., educational TV programs) to exert a positive effect on child language development, including (1) the match of the language in the program with child’s linguistic abilities; (2) children’s maturation in cognition (i.e., older than a toddler); and (3) the match of the content of the program with children’s comprehension level (Rice, 1983). The majority of studies that found the significant effects of multimedia quantity were based on experiments (e.g., Kuppens, 2010), and the language materials they have used were carefully selected to match participants’ current language ability. In contrast, the current study is an observational study and the participants have the freedom to choose whatever materials are available to them at home. Their multimedia input might be out of their zone of proximal language development, being beyond or below their proficiency level. Moreover, the materials could be more entertainment-oriented than education-oriented, resulting in a situation where the increased amount of language input leads to no substantial language improvement.

A larger number of resources, on the other hand, increased the chance of matching the multimedia input with children’s Mandarin proficiency level. Moreover, the diverse resources (e.g., games, eBooks, educational programs, and movies) provided children with rich vocabulary and linguistic structures to promote language building. Such language input could provide children sufficient language examples, which facilitates language entrenchment and abstraction (Lieven, 2019). Encountering the various types of exemplars in diverse contexts allows children to recognize the analogies between constructions (Bybee, 2006) and promote children’s language learning. In other words, a larger number of multimedia resources could offer bilingual children more authentic, rich, and complex heritage language input, which the children lack in conventional language settings.

## Conclusion

Multimedia is widely used in early childhood nowadays, and the current study focused on the effectiveness of the amount and number of resources of multimedia input on early bilingual language acquisition. The study found a differential effect on children’s societal dominant language and the heritage language. Multimedia exerted little influence on the former and showed significant effects on the latter for the English-Mandarin bilinguals in the current study. Simply increasing the quantity of multimedia input would not promote children’s heritage language learning, as it was the diversity of multimedia resources that has been found to significantly affect children’s Mandarin learning. Our finding is assumed to be important for multilingual societies like Singapore, where bilingualism is the foundation for its education. Parents in these countries usually prefer to speak the societal language to their children at home due to utilitarian concerns. They would probably rely on schools to develop children’s heritage language. Schools, on the other end, are busy with accommodating children with significantly different language proficiency levels in the same class. Teachers must work hard to optimize the limited instructional hours (40 min to 1 h per day on average) to provide children with good language input. Our findings provide another solution: a diversity of multimedia materials may be taken as supplementary input to the conventional sources (e.g., home and school), for effective early heritage language development. Tutorials should be provided to parents, to facilitate their multimedia selection and usage with their children.

There are four major limitations of the study. First, this is a cross-sectional study; therefore, only the relationship between multimedia input and children’s English and Mandarin language skills could be inferred. Future studies could follow the participants longitudinally and examine possible variations in the trajectory of the relations. Second, the current study only considers two general aspects of multimedia input, and future studies might further explore the effects of the specific features of multimedia (e.g., interactive questions, dictionaries, and music) on bilingual children’s dual language learning. Third, the current study has only tested three aspects of children’s vocabulary and grammatical skills, and future studies might employ an in-depth approach to assess children’s dual language proficiency. For instance, children’s vocabulary skills should not only be examined with labeling tasks but also with word description tasks to reflect their vocabulary depth. Last, the elicitation of home language input quantity and quality could be improved, as the parental survey could only generally reflect the home language environment and therefore not necessarily accurate. Future researchers could use the language diary approach (De Houwer and Bornstein, 2003) or Environmental assessing technology (e.g., LENA) to more precisely capture children’s language exposure with different interlocutors (e.g., with parents vs. with peers) and in different modalities (with interlocutors vs. using multimedia).

## Data Availability Statement

The datasets presented in this article are not readily available because: the data could not be shared due to PDPA. Requests to access the datasets should be directed to HS, he.sun@nie.edu.sg.

## Ethics Statement

The studies involving human participants were reviewed and approved by the IRB-2017-04-019, Nanyang Technological University Institutional Review Board. Written informed consent to participate in this study was provided by the participants’ legal guardian/next of kin.

## Author Contributions

HS designed the work, analyzed the data, and interpreted the results. HS and BY wrote the manuscript together. Both authors contributed to the article and approved the submitted version.

## Conflict of Interest

The authors declare that the research was conducted in the absence of any commercial or financial relationships that could be construed as a potential conflict of interest.

## Footnotes

1 1We recognize that some second language/bilingual researchers make a distinction between *uninstructed* acquisition and *instructed* learning (e.g., Krashen, 1981). Nevertheless, these two terms are sometimes used interchangeably in the literature (e.g., Housen and Pierrard, 2005), which is also the approach in this paper.

2 The children in the current study were early bilinguals, who have been exposed to both English and Chinese at home simultaneously or sequentially from birth. We excluded recent immigrants from China because such children are probably monolingual Mandarin speakers and their language experience would be substantially different from the rest of the population in our sample.
